# Some Facts on the Diabetes Surveillance in Rural Regions of Iran

**Published:** 2018-12

**Authors:** Ali Reza MAHDAVI HAZAVEH, Mohammad Hossein PANAHI, Elham YOUSEFI, Shahin YARAHMDI

**Affiliations:** 1. Ministry of Health and Medical Education, Tehran, Iran; 2. Dept. of Epidemiology and Biostatistics, School of Public Health, Tehran University of Medical Sciences, Tehran, Iran

## Dear Editor-in-Chief

Diabetes is one of the most important noncommunicable diseases, through its effects on many body organs. It is the major risk factor for cardiovascular diseases, kidney failure, blindness and increases risk of death. Incidence and prevalence of this disease have risen at increasing rate during the past decades (1). The number of people with diabetes will increase from 415 million in 2015 to 642 million in 2040 (2). The maximum prevalence of diabetes occurred in countries with low and average income levels (3).

Considering its increasing rate in the Eastern Mediterranean Region, it will rise from 9.7% in 2014 to 11.6% in 2040 (1, 4). The screening program for diabetes was implemented in the rural regions of Iran in 2005 and 2008 among the population aged over 30 years. Identification of patients has taken place opportunistically in rural regions since 2011 and the people with diabetes are entered into the country’s system of diabetes surveillance. In this program, patients were visited once every three months by a doctor and sent to laboratories for the HbA1c and fasting blood sugar tests. Results of the National Program for Prevention and Control of Diabetes in the Rural Regions in the second quarter of 2015 recorded in the portal of the Department of Non-Communicable Diseases at the Ministry of Health indicate that, among the 12 million rural population covered by these universities, at least 364,000 diabetes patients are under surveillance, and 67% of the male patients and 72% of the female patients have been taken care during this period.

About 74% of these patients had HbA1c levels less than 8, while 37% of these patients had HbA1c levels less than 7, and 50% of these patients had ideal fasting blood sugar levels (70–130) (Table 1).

**Table 1:** Results of diabetes surveillance rural population of Iran

***Sex***	***Population>30***	***Number of patients***	***Number of patients cared by GP***	***Number of FBS test***	***FBS 70–130***	***Number of HbA1C tests***	***HbA1C< 7***	***HbA1C< 8***
Male	6182836	110776	74124 (67%)	54286	27966 (52%)	23766	8765 (37%)	17630(74%)
Female	6050644	253477	181531(72%)	127915	62717 (49%)	54444	20562 (38%)	40172(74%)
Total	12233480	364253	255655 (70%)	182201	90683(50%)	78210	29327 (37%)	57802(74%)

* Data are shown as frequency (percentage)

In patients with diabetes, the treatment goal is to reduce HbA1c levels to 7% or lower. One percent decrease in HbA1C level reduces cardiovascular complications by 37%, myocardial infarction by 14%, and mortality resulting from diabetes by 21%. This indicates the importance of controlling the HbA1c level (5).
